# Patients’ Perceptions and Experiences With Outpatient Hip and Knee Arthroplasty: A Qualitative Study

**DOI:** 10.7759/cureus.78781

**Published:** 2025-02-09

**Authors:** Mark HF Keulen, Bert Boonen, Alexandra M Leenders, Emil H Van Haaren, Martijn GM Schotanus, Yoeri FL Bemelmans

**Affiliations:** 1 Department of Orthopedics and Traumatology, Zuyderland Medical Center, Heerlen, NLD; 2 School of Care and Public Health Research Institute, Maastricht University Medical Center, Maastricht, NLD; 3 Department of Quality Improvement, Zuyderland Medical Center, Heerlen, NLD

**Keywords:** outpatient hip arthroplasty, outpatient knee arthroplasty, patient experience, patient perspective, patient satisfaction, qualitative study, total joint replacement

## Abstract

Background

Outpatient joint arthroplasty (OJA) of the hip and knee is becoming increasingly popular within the orthopedic community. Most evidence regarding the safety and feasibility of OJA has been gathered from the perspectives of surgeons and healthcare organizations. However, the success of these pathways also hinges on patients feeling safe and adequately prepared to return home. This study aimed to explore patients' perspectives, experiences, and challenges during the preoperative phase, the day of surgery, and the postoperative phase of outpatient total hip arthroplasty (THA) or total knee arthroplasty (TKA). Patient satisfaction was also assessed.

Materials and methods

Twelve patients who underwent outpatient THA or TKA and their informal caregivers participated in face-to-face, semi-structured interviews. The transcripts were analyzed using thematic analysis, following the Grounded Theory approach.

Results

OJA was well received by patients, with 33% making a patient-driven decision to undergo the pathway, while the majority supported the idea following their surgeon's recommendation. Key areas for enhancing the patient experience included better preoperative education and expectation management, streamlined pathway logistics, and reassurance through robust intra- and extramural safety nets. Notably, 92% of patients indicated they would choose OJA again over an inpatient procedure if faced with the same decision.

Conclusions

Although patients were generally very satisfied with outpatient THA and TKA, the findings create scope to refine existing pathways further. Additionally, interview insights suggest potential improvements to patient selection criteria to identify suitable candidates for OJA better.

## Introduction

Background

Outpatient total hip arthroplasty (THA) and total knee arthroplasty (TKA) have gained popularity over the last decade. The feasibility and safety of these procedures have been repeatedly demonstrated in both selected and unselected patient populations [1-3]. Key drivers of this growing interest include continuous advancements in perioperative management, the implementation of evidence-based practices, more efficient use of resources amidst a near-unsustainable burden of joint replacements, and the ability to reduce costs within the episode of care [4-5]. The recent COVID-19 pandemic has further emphasized the trend toward risk stratification and outpatient joint arthroplasty (OJA). Efficient resource utilization, minimizing patient exposure to hospital wards, and reducing the strain on already overburdened inpatient beds became critical, especially as many elective surgeries (e.g., hip and knee arthroplasties) were intermittently halted, leading to dramatically increased waiting lists. In response, more OJA pathways were established in several clinics, enabling optimized hospital capacity management while safely discharging patients on the same day as their surgery [6-7].

A recent survey by Keulen et al. (2022) confirmed the growing interest in OJA among Dutch orthopedic surgeons. The survey revealed that approximately 20% had already established outpatient hip and/or knee arthroplasty pathways. At the same time, about half of the remaining surgeons expressed interest in implementing such pathways in the future [8]. Furthermore, the survey highlighted that patient demand was one of the primary reasons orthopedic surgeons adopted OJA. From this perspective, OJA can be described as “patient-driven.”

Most available evidence on the safety and feasibility of OJA has been gathered from the perspective of healthcare providers, including orthopedic surgeons and healthcare organizations. These studies typically evaluate success rates, complications, readmission rates, and cost efficiency [4-5]. However, a successful discharge also requires patients to feel safe and ready to return home without overburdening their informal caregivers. Orthopedic surgeons offering OJA should not overlook the principle of "first better than faster" [9], ensuring that patient-reported outcomes and experiences are prioritized to optimize the pathway. To date, however, only a limited number of studies have investigated the patient perspective regarding the implementation of OJA [10-12] or their experiences with it [12-16]. To the best of our knowledge, this is the first in-depth qualitative study to interview patients and their informal caregivers who have undergone outpatient THA or TKA.

Aims

We aimed to assess patients' perspectives, experiences, and challenges during the preoperative phase, the day of surgery, and the postoperative phase of outpatient THA or TKA. Additionally, we aimed to evaluate patient satisfaction with outpatient THA or TKA.

## Materials and methods

This qualitative study was approved by the Medical Ethical Review Committee of Zuyderland and Zuyd University of Applied Sciences (approval number: METCZ20200047). Verbal and written informed consent were obtained from all study participants.

Inclusion and exclusion criteria

All patients scheduled consecutively for outpatient THA or TKA were invited by a researcher to participate in this study. Inclusion criteria were patients aged 18 years or older, with an indication for elective primary THA or TKA; deemed eligible for OJA (defined as discharge to their own home on the day of surgery) according to the orthopedic surgeon (eligibility was primarily based on previous research by our group [17-19]); and fluent and literate in Dutch. Exclusion criteria included dementia or other medical conditions affecting the ability to provide verbal or written consent or reliably participate in the interview, as well as unwillingness to participate. Patients initially enrolled in the outpatient pathway but who failed same-day discharge (SDD) were not excluded from the study.

There was no financial or non-financial incentive for participation in the study. To minimize bias, all healthcare professionals involved in the OJA pathway were unaware of the enrollment period.

The sample size was determined using the principle of data saturation in qualitative research [20]. Recruitment ceased when both researchers (MK and YB) agreed that additional interviews no longer yielded new information or insights.

Setting and data collection

The study was conducted in the orthopedic department of a Dutch non-academic teaching hospital where OJA of the hip and knee was introduced in 2013 [17-18]. After obtaining informed consent, patients were invited to participate in a face-to-face, semi-structured interview. Patients were encouraged to bring their informal caregivers to the interview. Interviews were scheduled 7-14 days after surgery to minimize recall bias. The interviews took place in the orthopedic outpatient clinic and were conducted by one of two authors (MK or YB) accompanied by a research student. None of the interviewers were directly involved in the treatment of the participants. A topic list (Table 1) was developed prior to the study to guide the interviews. This list was divided into four main categories (preoperative phase, day of surgery, postoperative phase, and satisfaction), with subtopics selected based on expert opinions and researchers’ observations of the OJA pathway prior to the start of the study. Open-ended questions were included to provide a general structure for the interviews, and participants received the topic list in advance. Follow-up questions were used during the interview to explore participants' experiences in greater depth.

**Table 1:** Topic list OJA: outpatient joint arthroplasty

Topic	Subtopic	Sample questions
Preoperative phase	Decision-making	How was the option of OJA introduced during your preoperative consultation with the orthopaedic surgeon? What motivated your decision to undergo OJA?
	Preoperative expectations	What were your expectations of the OJA pathway? What were your thoughts on the feasibility of same-day discharge after surgery? How did you feel when deciding to undergo OJA? Did you experience any anxiety or fear?
	Education	How did you perceive the information provided to you prior to the OJA? How was the information delivered to you, and did you find it sufficient?
	Past experiences	Have you had previous experience with joint replacement or outpatient surgery? How did this influence your current decision? In what ways did your past experiences with joint replacement or outpatient surgery impact your journey through the OJA process?
Day of surgery	Short hospital stay	How would you describe your experience on the day of surgery?
	Burden	How would you describe the burden of the day of surgery for both you and your caregiver?
	Pathway logistics	Did you encounter any organizational or logistical challenges on the day of surgery?
	Discharge	How prepared and ready did you feel for discharge from the hospital?
	Anxiety/fear	What were your emotions during the day of surgery? Did you experience any anxiety or insecurity during the day or at the time of discharge?
Postoperative phase	Stay at home	How did you experience the first few days after returning home?
	Feelings and emotions	How would you describe your emotions after discharge (e.g., anxiety, fear, distress, uncertainty, loss of sleep)?
	Pain and medication	How would you describe your pain management after discharge? How did you find the adequacy and sufficiency of the medication provided after discharge?
	Safety net	How safe did you feel at home after being discharged from the hospital? Were there any times when your informal caregiver felt uncomfortable with the level of care required? How well were you informed about who to contact and when, in case of an emergency or adverse event? Have you ever needed to use any healthcare resources (other than your informal caregiver) for support after discharge?
	Adverse events	What adverse events, if any, did you experience after surgery?
Satisfaction	Meeting expectations	How did your experience compare to your preoperative expectations?
	Repeat	How likely are you to choose OJA again if it were indicated?
	Recommend	How likely are you to recommend OJA to others? Do you believe OJA is suitable for everyone? If not, what factors do you think are important for someone to be eligible for OJA?
	Tips/advice	What suggestions or advice would you offer to improve the OJA pathway?

Data analysis

**Figure 1:**
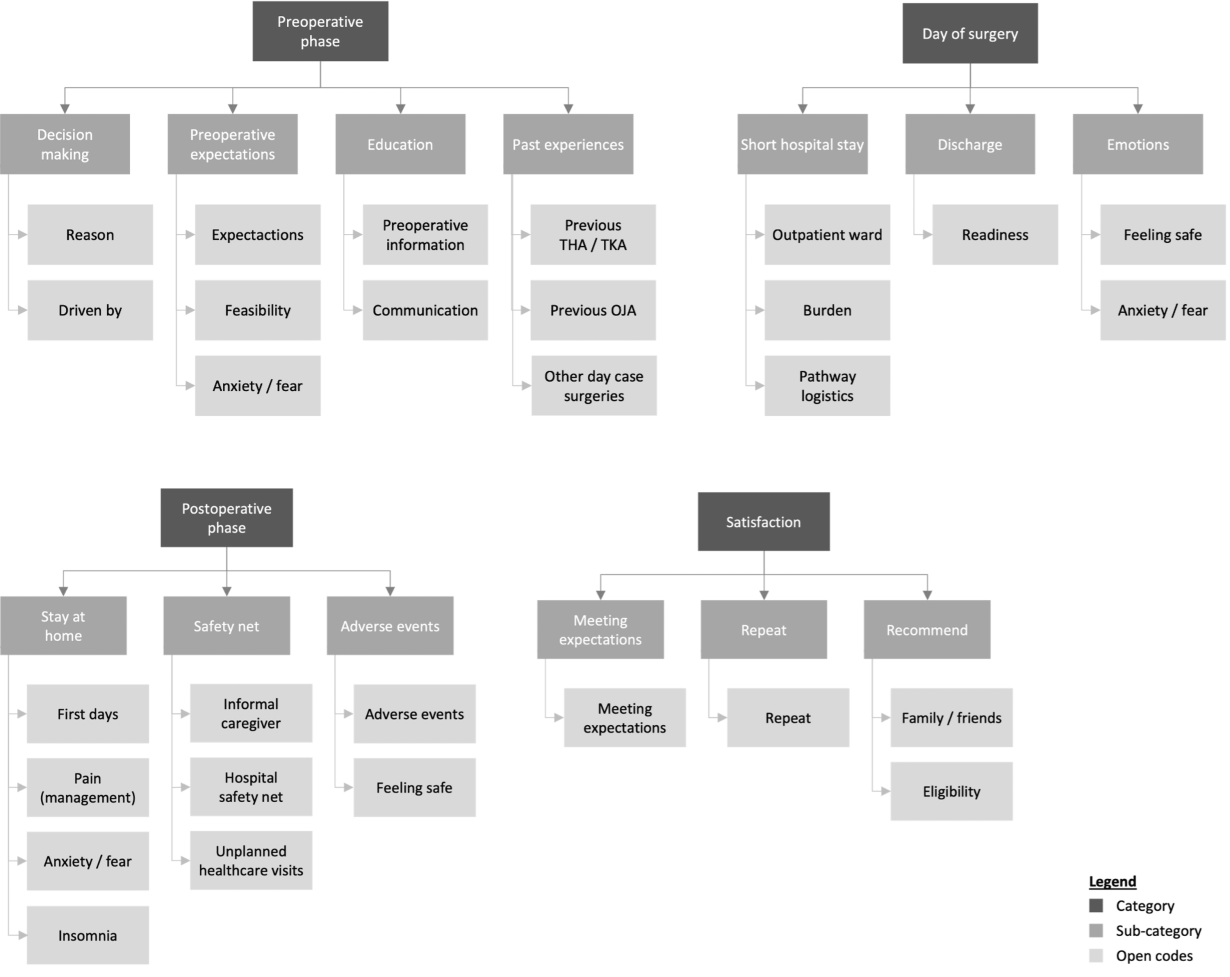
Code tree used for thematic analysis THA: total hip arthroplasty, TKA: total knee arthroplasty, OJA: outpatient joint arthroplasty

The interviews were tape-recorded and transcribed verbatim using oTranscribe (https://otranscribe.com). Thematic analysis of the transcripts was conducted according to grounded theory principles [21]. Categories (“preoperative phase”, “day of surgery”, “postoperative phase”, and “satisfaction”) and subcategories were partly predetermined based on the interview guide, so initial coding was deductive. Inductive coding was also applied, as categories were continuously refined based on the data analysis conducted by both the first author (MK) and the research students. The final list of codes is presented in Figure 1. Since the interviews were conducted in Dutch, all quotes were translated into English. A structured overview of quotes from all interviews is presented.

## Results

Demographics

A total of 12 patients and their informal caregivers were interviewed. The mean age of the patient cohort was 65 years (with a standard deviation of 7.5 years, ranging between 54 and 74 years) (Table 2). SDD was successful in 83%.

**Table 2:** Patient demographics P: patient, THA: total hip arthroplasty, TKA: total knee arthroplasty, SDD: same day discharge, LOS: length of stay

Patient	Sex	Age (in years)	Type of arthroplasty	Discharge	Highest educational level	Work (previous)
P1	Female	74	THA	SDD	Senior secondary vocational education	Retired (nurse)
P2	Male	71	THA	SDD	Prevocational secondary education (police training)	Retired (policeman)
P3	Male	56	TKA	SDD	Prevocational secondary education	Bus driver
P4	Female	67	THA	Unsuccessful SDD, LOS = 1 day	Higher vocational education (secondary teacher education)	Retired (secondary school teacher)
P5	Male	54	THA	SDD	Senior secondary vocational education	Electrical installer
P6	Female	72	THA	SDD	Higher vocational education	Retired (nurse)
P7	Female	70	TKA	SDD	University education	Retired (policy officer)
P8	Female	60	THA	SDD	Higher vocational education	Customer service employee
P9	Male	57	THA	SDD	Prevocational secondary education (police training)	Policeman
P10	Female	60	THA	SDD	Senior secondary vocational education	Personal health care assistant in elderly care
P11	Female	59	TKA	Unsuccessful SDD, LOS = 1 day	Senior secondary vocational education	Office worker
P12	Female	74	THA	SDD	Prevocational secondary education	Retired (hospitality worker)

Preoperative phase

Decision-Making

The orthopedic surgeon recommended undergoing OJA in eight cases based on the appropriate indication. However, most patients and their informal caregivers accepted the idea of SDD. The decision to undergo OJA was specifically requested by four patients, making it “patient-driven." The main reasons for choosing OJA included the surgeon’s recommendation, the patient’s level of fitness, the presence of a supportive informal caregiver, faster and better rehabilitation at home, the desire for more privacy, and the comfort of being surrounded by personal belongings (Table 3).

**Table 3:** Patients’ and caregivers’ statements regarding their experiences with the preoperative phase

P	Decision-making	Preoperative expectations	Education	Past experiences
1	“I just trust the surgeon on his words. I’m a layman, and I have no idea. I’ll just go with what he tells me.”	“I am not someone who overthinks things. I knew what to expect and felt confident that I could go home the same night as long as there were no complications.”	“The surgeon explained everything clearly during the consultation. I also got a bag with some brochures, which I read through. That was enough for me.” “The only thing is, I’d never used crutches before. It would’ve been nice if someone had shown me how to use them beforehand.”	“I have had a few day surgeries for other medical reasons before, so I think that helped me know what to expect."
2	“The surgeon mentioned day surgery to me, and I thought it sounded good to be able to go home the same day. After all, the hospital is for people who are really sick."	“I didn’t really mind it.” “At first, I was a bit unsure. I kept thinking, what if something goes wrong the first night after he gets home? Like if he had too much pain or the wound started bleeding. That made me nervous.” (caregiver)	“I got a folder with information. It could always be a bit more detailed, but it was enough for me.” “Also, I had already been researching it myself online.”	“I have had three-day surgeries before, but that did not necessarily help me prepare for this one. I’m just not someone who gets anxious.”
3	“I asked for it myself because I wanted to recover quickly, go home, and just do my own thing.”	“I was not afraid. I just wanted to have the surgery, recover, and go home once the doctors said it was okay.” “I thought it was a good idea too. I was not worried about it.” (caregiver)	“The information before the surgery could have been a bit clearer. I just got a bag with brochures, but I did not really find them helpful.”	“I have had surgery before for other things, and each time I just wanted to get home as soon as I could."
4	“The surgeon suggested it, and I agreed right away. My partner and I are both in good shape. If I had been on my own, I probably would not have done it.”	“I felt fit enough to go home the same day. As long as you have someone to help you at home, I thought it is definitely doable.”	“The information from the surgeon was good. I also got a brochure with all the details on rehab and some helpful tips.” “I felt well prepared.”	“I had surgery once before, but that was a long time ago. No, it was not really similar to this one.” “My sister told me about her two hip surgeries, but I guess everyone experiences it differently."
5	“The surgeon suggested it, but that was not an issue for us. We trust the surgeon's judgment.”	“At first, I never thought I would be able to go home the same day.”	“The surgeon explained the procedure and sketched the rehab process for me. I also got a brochure, but it did not have much info about the day surgery itself.” “At first, they even told me I would stay in the hospital for 2 to 3 days.”	“Not really.”
6	“I asked for it myself. The sooner I am home, the better.”	“I knew what to expect since I went through the same procedure on the other side. I didn’t see any issues and just assumed I would be able to go home the same day.”	“I was satisfied with the information the surgeon gave me.”	“Last year, I had the same surgery on my right side and was happy to go home the same day. I think that made this experience easier for us.”
7	“The surgeon told me that the usual stay was about two days, but same-day discharge was also an option. Personally, I think it is best to stay in the hospital for as little time as possible, as long as the surgeon says it is safe... I prefer being at home where I feel more comfortable and have my privacy, so that's why I chose to go home the same day.”	"At first, I was a bit nervous because last time I had to stay in the hospital for 3 days because my wound was leaking.” “...on the other hand, it felt great knowing I could go home the same day."	"The surgeon explained everything to me, and I got a brochure with a lot of information. I also did some research online myself. It was reassuring to know that I could stay the night if things didn’t go as planned."	“I had surgery on the other side before, so I had an idea of what to expect."
8	“Actually, day surgery sounded logical to the surgeon, so another option wasn’t really offered to me. He just mentioned that I could go home the same day.”	"Day surgery made sense to the surgeon. At first, I was not sure what to expect and felt like I had to figure it out on my own. It was a bit scary."	“I received a brochure with a lot of information, but theory is different from practice. They should have reassured us a bit more.”	“I had surgery a couple of times a long time ago, but this was different. Everything was new, and with something new, it's always a bit scary at first. You just don’t know what is normal and what is not.” “Other people I know had undergone joint replacement in day surgery before, but you know, everyone is different.”
9	“The surgeon gave me the option to go home the same day.” “There's no place like home. I feel more comfortable recovering at home, away from the hospital environment where you are exposed to a lot of external impulses."	“I just hoped the pain would be better. I did not have many other expectations, really. I sort of went into the program without much in mind."	“The information we got was more than enough. If there was something I didn’t understand, I would just look it up online. There's so much information out there." "Before the surgery, I made sure to stay as active as possible and ate a healthy diet. I think that really helped with my recovery, and I would recommend it to anyone."	"I had not had surgery before, so I was not sure what to expect."
10	"The surgeon suggested I go home the same day, and I was fine with that. Honestly, I don’t like being in the hospital, and I thought I would recover faster at home. Plus, if anything went wrong, I knew I could stay longer if needed."	"I had surgery on the other hip three years ago, so I expected it to be pretty similar. I figured I could handle going home the same day."	“I felt that the information I got before the procedure was not enough. The brochure I received did not have any details about day treatment specifically." "Three years ago, there was a meeting before the surgery where they checked the crutches and showed some exercises. I missed that this time, as I found it really helpful."	“When I had my other hip replaced three years ago, I had to stay in the hospital overnight and went home the next day.”
11	“The surgeon suggested same-day discharge, and I thought that was normal, so I agreed. If I had been given the choice to stay a few days or go home the same day, I probably still would have chosen to go home because I prefer recovering at home rather than in the hospital.”	“Sometimes I think it is a bit ambitious to go home so soon after an operation. But I guess I wasn't the first, and I definitely won't be the last."	“The information I received was great. I didn’t feel like I missed anything.”	"I have had day surgery before, but for something different. It didn't really help me know what to expect this time."
12	“I agreed to the surgeon's suggestion for day surgery. I am not a fan of being in the hospital, so if I'm feeling okay and things are going well, I would rather go home as soon as possible.” “Staying in the hospital will only make you sicker.”	“I had no doubts about the program. I just expected that if I felt good, I would be able to go home the same day." "Since I'd had surgery on the other hip before, I knew what to expect."	“The information I received was more than enough. Having gone through the procedure a few years ago, I already knew what to expect."	“After my previous operation, I stayed in the hospital for 6 days. I understand that some people prefer to stay and be pampered in the hospital, but personally, I preferred to go home as soon as I could."

Patient 4: “The surgeon suggested it, and I agreed right away. My partner and I are both in good shape. If I had been on my own, I probably would not have done it.”

Preoperative Expectations

Although 67% of patients had expectations aligned with the OJA pathway as described and just expected to be discharged home on the same day without any issues, some patients had no specific expectations. However, two patients found the idea of SDD ambitious, with one of them even doubting its feasibility at first. Additionally, two patients and two informal caregivers expressed concerns, fearing the unknown and the loss of control (Table 3).

Informal caregiver 2: “At first, I was a bit unsure. I kept thinking, what if something goes wrong the first night after he gets home? Like if he had too much pain or the wound started bleeding. That made me nervous.”

Education

Patients primarily received information through face-to-face communication and informational brochures, with 25% reporting self-research online for additional details. Four patients were not entirely satisfied with the information they received: two felt the information was too impersonal, and three suggested a more detailed explanation of the OJA pathway. Patients agreed that better information provision and practical tips through face-to-face joint school sessions could have alleviated some concerns about the surgery and the outpatient pathway (Table 3).

Patient 5: “The surgeon explained the procedure and sketched the rehab process for me. I also got a brochure, but it did not have much info about the day surgery itself.”

Past Experiences

Five patients reported a history of contralateral joint replacement, one of whom had previously completed the OJA pathway. According to most patients, this experience helped manage their expectations and reduce preoperative anxiety (Table 3).

Patient 6: “Last year, I had the same surgery on my right side and was happy to go home the same day. I think that made this experience easier for us.”

Day of surgery

Short Hospital Stay

All patients were generally satisfied with the short hospital stay. Subsequent care was well-coordinated in most cases, and while some found the day long, none felt it was overwhelming. Most patients valued their autonomy in rehabilitation and were pleased with their rapid progress. However, two patients felt they could have been discharged earlier due to extended periods of waiting or inactivity and suggested that physical therapy could be more intensive. Additionally, three patients reported long waiting times (two to three hours) on the ward before surgery, and medication dispensing logistics were inadequate in four cases because of delays at the hospital pharmacy (Table 4).

**Table 4:** Patients’ and caregivers’ statements regarding their experiences with the day of surgery

P	Short hospital stay	Discharge	Emotions
1	“It was a day that went by smoothly.”	“I felt safe and ready to go home.”	’I was not scared at all. I have had surgery plenty of times before, and I knew my daughter would be there to help me if I needed anything the first few nights."
2	“You know what’s going to happen and that you’ll be able to go home at the end of the day.” “Because we stayed longer and the nursing staff changed, they almost forgot to take the X-ray. The communication could have been a bit smoother." “I was expecting more practical advice from the physical therapist or nurse to help my husband, especially for things like getting out of bed or going to the bathroom." (caregiver)	"After the surgery, the numbness lasted for a long time, so I couldn’t do much with physical therapy initially. Since I was expected to go home the same day, I would have preferred if I had my strength and feeling back sooner. Now we were just waiting there. Eventually, however, I was ready for discharge.”	"I wasn’t afraid or anxious at all. I went into the surgery feeling very confident."
3	“The day of surgery went smoothly overall. My partner just dropped me off and picked me up. The only issue was having to wait a long time at the hospital pharmacy for the discharge medication. I understand it can be busy, but after surgery, the wait feels uncomfortable. Why not prepare the medication in advance and bring it to you on the floor?"	“I felt like I could have gone home two hours earlier than planned, but I had to wait for the last dose of antibiotics. I think I was ready to leave before the schedule allowed it.”	“I was relieved to go home. I was not scared at all.”
4	"Everything was well organized until the point when I needed to stay overnight.” “Even though it was clear I had to stay overnight, I was not logged in the system as an active patient, so I was not included for breakfast, the X-ray was not scheduled for the next day, and getting my discharge medication from the pharmacy did not go smoothly either. That was frustrating."	"The surgeon reassured me that the surgery went well, but I had to stay overnight as a precaution because of some blood loss. I thought this was a good idea. It felt safer to stay in the hospital for a night rather than risk going home and facing any complications that you can’t foresee. The next day, I also felt ready to go home." "As a caregiver, I think it’s important to consider not just physical fitness but also stress levels before discharge. It's essential to make sure the patient is mentally ready to handle rehabilitation at home." (caregiver)	“I felt a healthy dose of tension at the time of discharge.”
5	“You can’t say anything bad about it. The healthcare professionals were supportive and made me feel confident throughout the day.” “The day itself felt calm. I was happy to stay, receive tips and tricks advice, and help guide him throughout the day.” (caregiver)	“It all felt good to go home.”	"I wasn’t scared at all, even when the physical therapist tried to get me out of bed for the first time, and I felt dizzy." "I'm a nurse myself, so I'm used to these situations. I don’t get scared easily." (caregiver)
6	“It all went very smoothly. The whole process felt very professional, and that was reassuring." "That day, we realized we were a bit lucky. The surgical schedule was partly blown off because of COVID. Since I had surgery early in the morning and was scheduled to go home the same day, it didn’t affect me. It just gives another reason to promote day surgery."	“I 100% agreed with the doctor and physical therapist and felt ready to go home.”	I did not feel overwhelmed or anxious during the day or when it was time to go home." "We're both not the anxious type. Plus, we've both worked in the hospital for a long time." (caregiver)
7	"When I had surgery on the other side, rehab with the physical therapist went a lot slower and was more gradual..." "Now, I actually liked the intensity of the day. At least there was something to do, and it felt more suited to my level. Otherwise, you're just lying in the hospital bed all day.”	“There was a bit of bleeding from the wound, but the nurse took care of it, and after that, I was ready to go home.”	"I didn't feel scared at all. I wasn’t worried about the wound leaking again either."
8	“I had to be there at 7:15 AM, and I didn't go to the OR until 10:00 AM. Why do you have to be there 3 hours in advance? That waiting time could have been shorter.” “The day went well overall. I appreciated how active they were with walking and rehab. I just wish they had addressed some of my concerns before the surgery.”	“I was in the hospital for about 13 hours, knowing I would be going home. When it was time to leave, I felt good enough and safe to go." "However, if I had been given the choice before the surgery, I probably would have chosen to stay the night."	"I was a bit worried about the first night and the morning after. I thought it would have been helpful if the hospital staff had provided more guidance and reassurance, like ‘this is the best way to do this and that.’ In my opinion, that's a downside of day surgery."
9	“It was quite a long day. In my opinion, the waiting time from when I arrived at the hospital until the surgery was too long. It could have been shorter since the preparations were minimal.” "After surgery, I liked the quick movement and exercises with the physical therapist."	“The doctor, physical therapist, and nurse all said I was ready to go home, and I felt the same way.”	“I was not scared at all. I was really looking forward to the surgery and getting home afterward.”
10	"The first time the physiotherapist gets you moving always feels too soon, but once you start and see that things are going well, you quickly feel more confident."	"I felt ready to go home by 1:00 PM, and the physical therapist agreed. But we had to wait until 4:00 PM for the ward doctor to clear me. It could have been more efficient, but I guess there are things happening behind the scenes we don’t know about."	"I wasn’t worried at all during my short stay or when it was time to go home.”
11	"By the end of the day, I still had no feeling or strength in my legs, so I couldn’t do therapy and had to stay overnight."	"The next day, I was able to go home. If the anesthesia had worn off sooner, I probably could have gone home the same day."	"I was not worried, even though the feeling in my legs did not come back at first. I knew I was in good hands."
12	“Therapy in the hospital was too limited. They just walked with me down the hallway and up the stairs, and then I went back to my room…” "The whole day felt too long. I was already trying to figure out for myself when I could go home."	“I felt comfortable going home.”	“If I did not feel comfortable going home, I would have told the doctor that I was worried. But I was not.”

The two patients admitted to the inpatient ward after unsuccessful SDD reported issues with the quality of handover and internal communication between wards and disciplines. These included being overlooked for meals, miscommunication about medications, and scheduling challenges for postoperative X-rays.

Patient 2: “Because we stayed longer and the nursing staff changed, they almost forgot to take the X-ray. The communication could have been a bit smoother."

Patient 7: “When I had surgery on the other side, rehab with the physical therapist went a lot slower and was more gradual..." "Now, I actually liked the intensity of the day. At least there was something to do, and it felt more suited to my level. Otherwise, you're just lying in the hospital bed all day.”

Discharge

All patients successfully discharged on the day of surgery reported feeling safe and ready for discharge. Three patients noted that they felt ready even earlier than planned. Two patients, however, were not deemed medically fit for SDD and required overnight stays - one as a precaution due to 1000 mL intraoperative blood loss and the other because of weakness and numbness that impaired early ambulation (Table 4).

Patient 3: “I felt like I could have gone home two hours earlier than planned, but I had to wait for the last dose of antibiotics. I think I was ready to leave before the schedule allowed it.”

Emotions

Eleven patients reported no fear or uncertainty during the day of surgery. However, one patient and one informal caregiver expressed slight uncertainty about the first night at home (Table 4).

Patient 8: "I was a bit worried about the first night and the morning after. I thought it would have been helpful if the hospital staff had provided more guidance and reassurance, like, ‘This is the best way to do this and that.’ In my opinion, that's a downside of day surgery."

Postoperative phase

Stay at Home

Most patients found their pain levels acceptable, recognizing it as a normal response to surgery. However, two patients reported significant pain during the first few postoperative days. All 12 patients stated that the prescribed pain medication was sufficient or more than adequate. In addition to regular paracetamol (1000 mg, four times daily) and meloxicam (15 mg, once daily), eight patients required rescue medication (OxyNorm® Instant 5 mg) at some point at home. Three patients (25%) experienced insomnia and suggested the temporary addition of benzodiazepines to the postoperative medication regimen. Only one patient reported mild anxiety related to postoperative swelling in the leg, which resolved without intervention (Table 5).

**Table 5:** Patients’ and caregivers’ statements regarding their experiences with the postoperative phase

P	Stay at home	Safety net	Adverse events
1	“The first couple of days, I couldn’t do much because of the pain, but the medication helped. I wasn’t worried because I knew it was surgery-related pain.”	“The physiotherapist visited me at home to instruct me and was able to answer my questions. If something unexpected had happened, I probably would have called my GP. Plus, I had an emergency number from the hospital, just in case."	“Nothing unexpected really happened. I did have some swelling in my leg, but the physiotherapist assured me that it was normal after this kind of surgery.”
2	"The only thing I might have done differently if I had stayed in the hospital is ask the doctor for a sleeping pill. I always struggle to sleep on my back." "I feel like it might have been better if he had stayed in the hospital overnight. The nurses could probably have managed his pain complaints better." (caregiver)	"I felt like we had a safety net because the nurses gave us clear instructions and phone numbers and told us when to call if needed." "The first night, he was in a lot of pain and did not sleep well. All I could do was give him the painkillers, but I’m not a nurse, so I felt a bit helpless." (caregiver)	"Other than the pain in the first few days, everything went as expected."
3	"I didn’t sleep the first night, but I also didn’t take any painkillers. The second night, I took Oxycodone and slept like a baby. It’s still surgery, so I guess some pain is normal. Other than that, the recovery went smoothly, and I was not worried."	“For the small wounds on my leg, I went to the family doctor because the physical therapist advised me to do so. I felt no need to contact the hospital, and the family doctor was only 100 meters away from where I lived. I knew, however, that I could contact the orthopedic department in case of emergencies.”	"The two small wounds on my lower leg got infected, so my family doctor prescribed antibiotics."
4	"Everything went well, I only had mild pain, and I was able to walk with crutches fine. I did have some swelling in my leg. Then I started to question everything: Is this normal? Should I rest more? Or should I be doing more exercises? It would’ve been nice to have some reassurance or tips from a professional." "I was glad I had someone to help me at home. I must admit, I felt a little emotional at times, wondering if everything would turn out fine since the rehab process is long. It was comforting to share that with my partner and go through it together."	"We didn’t feel left in the dark at all. We even called the orthopedic department a few days after surgery with a question about the wound, and they were able to reassure us right away." "As a caregiver, you spend most of the day taking care of him. We were wondering, how do people who live alone do all this?" (caregiver)	“No.”
5	“I had some swelling after surgery, but I knew that was normal. Each day, it got a little better.”	“I felt like I could always call or go to the hospital if there was an emergency.”	“We had no problems. It all felt very good.”
6	"We were able to manage everything on our own with the tips and medication they gave us at the hospital. I didn’t even need the oxycodone."	"We knew who to reach out to if anything went wrong. The hospital also called the next day to check on me, which made me feel more comfortable."	"Luckily, everything went well. That's really the only thing you might feel anxious about."
7	“I did not feel like I needed extra help from the nurses or physical therapists. The pain was manageable, I did my exercises, and I didn’t have any other issues like nausea. I think we managed just fine on our own.”	“The most important thing was having my partner with me at home. I also knew who to call if something went wrong. A few days later, the department called to check in and see how I was doing. I liked that.”	“Everything went fine. There were no problems.”
8	“At home, I felt a bit dizzy and nauseous at first, but it only lasted for a day, and I thought that it was probably part of the surgery. The pain was manageable with the medication.”	“I didn't like that they hadn't called the day after surgery." "On Sunday, we went to the emergency department. It was really reassuring to know that professional help is available 24/7." (caregiver)	"Over the weekend, I visited the emergency department because of pain in my knee on the same leg. The orthopedic surgeon examined me, and the X-rays came back fine. That was reassuring."
9	"We haven't really had any big issues or moments of worry."	"We didn't really miss help from the medical staff in the first few days after surgery. We knew who to contact if anything came up. Besides, they called me a couple of days later to check in and see how I was doing."	“None.”
10	"The first night went fine, but overall, I had more pain, and rehab was harder for me compared to last time. That was a bit disappointing."	"The next day, my leg was swollen, and I felt a bit feverish. My daughter called the outpatient clinic, and they said they would discuss it with the doctor and call me back, but I never received that call."	"Other than my leg being swollen, I didn’t have any issues. After resting, it felt better the next day, which was reassuring."
11	"Every day I noticed some progress in my recovery, so everything went well. We were also able to manage the pain on our own with the medication provided."	“I couldn’t have done it without my caregiver. Especially the first couple of days after surgery.” "We felt safe at home and knew who to contact if anything went wrong. Plus, the physical therapist came by, checked everything, and gave us exercises. It was reassuring because they have a good sense of what is normal and what’s not." "I took care leave from work. But seeing how she improves every day, I think she’ll manage just fine when I go back." (caregiver)	“No, it went really well.”
12	"The day of surgery went fine, but the next day at home, the pain really kicked in." "I would have appreciated it if they had given me something to help me sleep. Not being able to sleep makes it even harder to deal with everything." "The pain was manageable on my own, and I don’t think staying in the hospital would have added any benefit."	"You definitely need a caregiver to help with daily activities and personal care for the first few days after surgery." "If there had been an emergency, I would have called the emergency department." "I felt confident enough to assist her.” (caregiver)	"Not really, but since the surgery, I have had some nausea and headaches. I guess this is pretty common after such a major surgery."

Patient 11: "Every day I noticed some progress in my recovery, so everything went well. We were also able to manage the pain on our own with the medication provided."

Safety Net

All interviewed patients had an informal caregiver at home, and seven explicitly emphasized that having an informal caregiver is essential for successful OJA. Only one informal caregiver experienced a moment of discomfort due to the patient’s pain level. Eleven patients (92%) expressed satisfaction with the safety net provided by the hospital. Two patients (17%) required unplanned healthcare visits - one to the family doctor and one to the emergency department - and two patients (17%) contacted the outpatient clinic with questions (Table 5).

Informal caregiver 2: "The first night, he was in a lot of pain and did not sleep well. All I could do was give him the painkillers, but I’m not a nurse, so I felt a bit helpless."

Informal caregiver 4: "As a caregiver, you spend most of the day taking care of him. We were wondering, how do people who live alone do all this?"

Adverse Events

Four patients reported minor adverse events, including superficial wound infection, nonspecific knee pain following THA, headache and nausea, and postoperative fever of unknown origin. Patients who experienced these events while at home sought care for treatment or reassurance. However, they did not feel that staying in the hospital would have provided a significant advantage in managing these issues (Table 5).

Patient 6: "Luckily, everything went well. That's really the only thing you might feel anxious about."

Satisfaction

Meeting Expectations

Patients’ experiences aligned with their expectations in 42% of cases. Three other patients (25%) were pleasantly surprised by how quickly they recovered, noting that the process was smoother than anticipated. Similarly, three others, initially hesitant about the feasibility - perceiving SDD as too ambitious or feeling anxious about it - were ultimately satisfied and positively surprised with the result. However, one patient expressed mild disappointment, stating they had underestimated the intensity of the OJA experience (Table 6).

**Table 6:** Patients’ and caregivers’ statements regarding their overall satisfaction with the pathway

P	Meeting expectations	Repeat	Recommend
1	"I knew what to expect, so yeah! You just need to stay positive and go with the flow."	“I would prefer day surgery again for a next time. I like to be home and do whatever and whenever I want. I love the privacy.”	“Absolutely!”
2	"For me, everything went as I expected. It was also helpful that I had discussed the process with my physiotherapist beforehand."	"I would not want it any other way. If the other hip needs surgery, I’d choose the same surgeon and go for day surgery again." "Even though I had some worries at first, I’d definitely recommend day surgery again next time." (caregiver)	"No one believed I had surgery in the morning and was able to go home the same day. I would recommend the same to anyone." "Yesterday, we had a friend visiting from Germany, and she couldn’t believe it. She said in Germany, patients would stay in the hospital for 1-2 weeks." (caregiver)
3	"I recovered faster than I thought I would."	"Yes, I would definitely choose day surgery again if needed."	“Yes, I would definitely recommend it to others.” "I can understand if someone needs to stay longer, especially if they are older or do not have someone to help at home."
4	"If the surgeon had not told me about the excessive blood loss during surgery, I probably would have gone home the same day."	"Yes, I would do it again, but only if the surgeon thinks it is a good option."	"Yes, but only if the patient has a caregiver at home who is fit enough to help."
5	"Before surgery, I never thought I'd be able to go home the same day, but everything went well, and I could see the progress each day. In the end, going home was the best option for us." “We could say it actually went better than expected.” (caregiver)	“I would do day surgery straight again.” “Yes, now we know the procedure and what to expect.”	"Yes, I would definitely recommend it to others and suggest they follow the surgeon’s advice."
6	“It went exactly as we had imagined it before surgery. It was very similar to last year when I had surgery, but this time, it felt a bit smoother and easier."	“I already did it again, and I wouldn’t have it any other way.”	"I’m going to recommend day surgery to everyone, but I think it’s important that people are motivated to participate in such a program. Besides, having a caregiver at home is crucial. If you are all alone, it’s a whole different story.”
7	"I’m actually more positive about it now than I thought I would."	“I've already had both of my knees replaced, but if I ever need a hip replacement or something similar, and going home the same day is an option, I would definitely go for it.”	"I would recommend it to others, but it really depends on the person. They should be in good shape, motivated, and have someone available at home to help them."
8	"In the end, the day treatment went really well and was not as bad as I thought. But, of course, anything unknown can be a little scary at first. I think I just needed to experience it myself."	“If my caregiver and I are still fit, I would prefer day surgery. Now I know all the ins and outs.”	"I wouldn’t strongly recommend it to someone, but I would share my experience and give my opinion. Ultimately, it's up to them to decide what is best for them."
9	"As I mentioned earlier, I didn't have any specific expectations, but I was really happy with how everything turned out."	“We don’t see any downsides to same-day discharge.” “I would do it again. The less time spent in hospital, the better!”	"Yes, I would recommend it to everyone, but I think they need to be healthy, motivated, and resilient."
10	“It was harder than I expected. I thought I could handle it easily, but maybe I underestimated it. I should have prepared myself better and read more about it.”	"If I had to choose again, I’d probably stay overnight. I think I overthink too much, and maybe staying longer would help me find my internal peace and recover more smoothly."	"It really depends on the person, but in general, I would recommend it to family or friends who I think are capable of handling it."
11	"Even though I had to stay in the hospital, I was really satisfied with the treatment." "At first, I thought the idea of going home the same day seemed a bit ambitious, but in the end, I wasn’t disappointed."	"Now that I know what to expect. Everything is going really well, and based on that, I would definitely choose it again.”	“Yes, yes, yes!”
12	"Everything went as expected, and there were no complications." "I always try to stay positive and don't worry about tomorrow because it will bring its own challenges."	“In case I need another hip operation, it will be a day treatment again, as far as I’m concerned.” "Even if I were 80 and felt like I do now, I would still prefer to go home the same day. Age is just a number."	“Everybody is different, but If they are open to it, then I would definitely recommend it.”

Patient 10: “It was harder than I expected. I thought I could handle it easily, but maybe I underestimated it. I should have prepared myself better and read more about it.”

Repeat

The majority of patients (92%) indicated that they would prefer SDD over an inpatient procedure if they had to undergo THA or TKA again (Table 6).

Patient 9: “I would do it again. The less time spent in hospital, the better!”

Recommend

All 12 patients stated that they would recommend OJA of the hip or knee to family, friends, and relatives. However, they emphasized that eligibility for OJA depends on individual circumstances. Patients highlighted intrinsic motivation, self-reported vitality (regardless of age), and the availability of an informal caregiver as critical factors for a successful outcome (Table 6).

Patient 12: “Everybody is different, but if they are open to it, then I would definitely recommend it.”

## Discussion

OJA represents a natural evolution in joint replacement pathways, primarily driven by clinical and economic considerations. Until now, most efforts have focused on optimizing the perioperative phase from the healthcare provider's perspective to achieve favorable outcomes [2-5]. However, while patients play a central and active role in the entire process and stand to benefit the most from such optimized pathways ("first better than faster"), their perspectives as care recipients remain underexplored. With the successful reduction of length of stay (LOS) to SDD, a significant portion of care shifts from intramural (the hospital) to extramural (home). This shift highlights the importance of identifying common challenges patients and their informal caregivers face - from the preoperative consultation and preparation to the (non-)medical issues that may arise in the first postoperative days at home.

Preoperative phase

We found that 67% of patients' preoperative expectations were met with the OJA pathway described, and only 58% were satisfied with the information they received preoperatively. These findings suggest a gap in patient education and expectation management, which in some cases may have contributed to preoperative fear of losing control and hesitation about the feasibility of SDD. The discontinuation of preoperative joint classes during the course of this study due to the COVID-19 pandemic may be one possible explanation for this gap. To improve the current pathway, patients suggested adding specific informational materials (e.g., brochures) that outline the OJA pathway, including the benefits and risks of SDD, their role in a successful outpatient experience, discharge criteria, and the availability of an in-hospital backup plan in case SDD is unsuccessful. A recent study by Tolk et al. (2021) emphasized the importance of enhanced preoperative patient education, concluding that it can help modify patient expectations, leading to higher postoperative fulfillment of expectations and greater satisfaction [22]. In our study, postoperative fulfillment of expectations occurred in about half of the patients, while most others were positively surprised by the outcome of OJA.

Interestingly, we found that for patients who preoperatively reported fear or had mismatched expectations, the decision to undergo OJA was predominantly recommended by the surgeon ("surgeon-driven"). This highlights the importance of recognizing that OJA may not suit everyone. In an online crowdsourcing survey, the majority of the public preferred inpatient TJA, with only a minority expecting to be discharged home the same day [23]. A study by Adelani and Barrack (2019) revealed that approximately 70% of patients did not believe they could undergo TKA as an outpatient [10], a finding also supported by Meneghini and Ziemba-Davis (2017), who showed that in an unselected patient population, only 34% felt comfortable with the idea of SDD after THA or TKA [11]. In line with these studies, our patients indicated that intrinsic motivation was a critical factor for success in addition to overall good health and a supportive home environment (Table 3). In addition to existing selection criteria [19], surgeons should consider these factors when consulting with patients. We recommend shared decision-making to optimize patient autonomy and ensure the best possible outcomes.

Day of surgery

Although the day of surgery can often be perceived as "timed and intense for patients" by healthcare professionals, our results showed that patients viewed it as "a long day, but easy to handle." Patients were generally satisfied with the day of surgery. Despite the relatively short stay, many chose to shorten it further due to long waiting times before and after surgery, which they deemed "unnecessary." This again underscores the idea that outpatient surgery and reduced LOS can be "patient-driven." Equally important, we found no discrepancy between patients' and healthcare professionals' perceptions of safety and readiness for discharge from the hospital [24]. However, one patient and their informal caregiver expressed some fear, felt helpless, or lacked guidance during the first days at home. Interestingly, this patient had initially hesitated to undergo OJA, and the decision was largely "surgeon-driven." With OJA, the time patients spend with healthcare professionals postoperatively is shortened, which means that patients and their informal caregivers assume more autonomy and responsibility during rehabilitation. This includes managing pain and other postoperative discomforts, as well as coping with setbacks. To better select ideal candidates for OJA, we believe it may be beneficial to expand the selection criteria to include factors such as self-efficacy, resiliency, effective coping strategies, and mental well-being [25-27].

Our findings also emphasize the need for continuous improvement in pathway logistics to reduce patient burden and enhance their experience [9,28]. Patients suggested several improvements to our pathway, including better handover communication between outpatient and inpatient nursing wards and reducing unnecessary bot waiting times both before and after surgery. Suggestions included reducing waiting between physical therapy sessions, taking postoperative X-rays in the post-anesthesia care unit, and dispensing discharge medications directly on the ward (Table 4).

Postoperative phase

The shift from intramural to extramural care can evoke concerns among both patients and healthcare professionals regarding the safety of mobilization, pain management at home, and potential complications [10-11,14,23,27-28]. This underscores the importance of a robust intramural safety net, ensuring that patients can be admitted to the nursing ward if necessary, as well as an effective extramural safety net, where all parties involved (e.g., emergency department, general practitioners, and district nurses) are well-informed about possible complications [9].

Our findings also highlight the critical role of the social safety net, with the informal caregiver (coach) serving as an essential link throughout the entire pathway. Most patients reported a normal postoperative course, consistent with the recovery patterns described for THA by Klapwijk et al. (2017) [29] and TKA by Van Egmond et al. (2015) [30], and anxiety did not play a major role. This aligns with Hoorntje et al.'s (2017) findings that OJA does not negatively affect outcomes related to anxiety, depression, satisfaction, or pain levels [26].

Interestingly, some patients even believed their recovery was faster in their home environment compared to the hospital, a sentiment consistent with Adelani and Barrack's (2019) findings [10]. Four patients experienced adverse events and sought care. The frequency of unplanned healthcare episodes was comparable to those reported in the literature [1-2,13]. Of these, only one was considered a complication (superficial wound infection), while the others were primarily related to anxiety over self-limiting postoperative symptoms. Since patients reported that they would not have been better off in the hospital during these events, these results underscore the safety of OJA from the patients’ perspective (Table 5).

Satisfaction

Overall, patient satisfaction was very high, with more than 92% of patients stating they would prefer OJA if the contralateral limb required THA or TKA and 100% indicating they would recommend OJA to family and friends. These findings align with other studies that also report high satisfaction rates with OJA, which may even surpass inpatient pathways in terms of patient satisfaction. OJA may enhance the patient experience by providing dedicated postoperative support, reducing postoperative inconveniences, optimizing pain management, allowing patients to return home sooner, and facilitating faster functional recovery (Table 6) [12-16].

Strengths and limitations

This study's results should be interpreted considering its strengths and limitations. While existing literature emphasizes favorable patient experiences with OJA [12-16], this study is the first to conduct in-depth qualitative interviews shortly after patients underwent outpatient THA or TKA. Unlike prior research, which often focuses exclusively on successful cases [12,14], our study also included patients (and their informal caregivers) for whom planned SDD was unsuccessful, providing a more representative population. However, as interviews were conducted one to two weeks post-surgery, there is a potential risk of recall bias, particularly concerning preoperative sentiments.

The distribution of patients undergoing THA and TKA presents a potential limitation, as we recognize the inherent differences in recovery between these procedures [29-30]. Nevertheless, we found no significant differences in patients’ perspectives and experiences across both pathways, enabling data saturation and supporting our ability to answer the primary research question. Additionally, the study sample size aligns with norms for qualitative research of this type.

Our study found success rates for SDD comparable to those reported in the literature [2,13,17], and the study population displayed good diversity in patient characteristics. However, this study was conducted within the orthopedic department of a large non-academic teaching hospital with significant experience in OJA and pathway optimization, which may limit external validity. This setting differs substantially from many ambulatory surgical centers, where the threshold for inpatient admission may be higher.

## Conclusions

From the patients' perspective, outpatient THA and TKA were generally well accepted. However, the decision to undergo the OJA pathway should be mutual between the caregiver and care receiver, as OJA may not be suitable for everyone. While patients reported high satisfaction with OJA in a well-established pathway, their feedback highlights the ongoing need to refine the pathway to ensure both high-quality and patient-centered care. Key areas that could improve the patient experience include strengthening preoperative education and expectation management, providing reassurance through the availability of both intramural and extramural safety nets, and reducing patient burden by streamlining pathway logistics.
